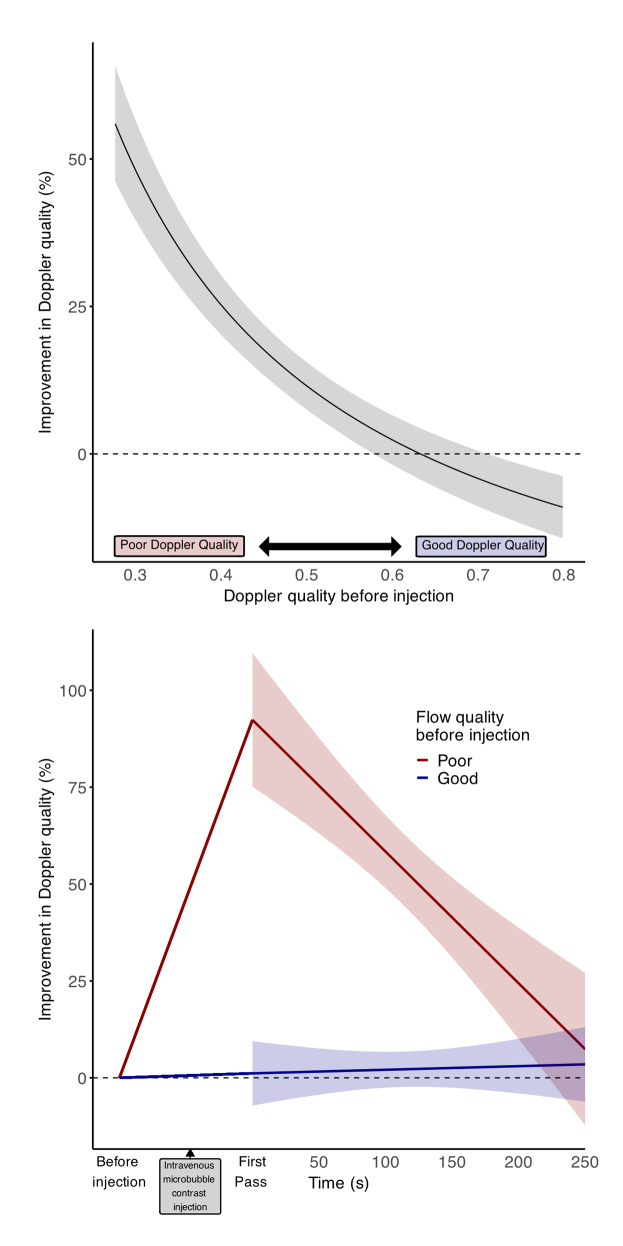# Microbubble Contrast Agent Use During Invasive Coronary Microvascular Assessment

**DOI:** 10.1016/j.jscai.2025.103857

**Published:** 2025-07-31

**Authors:** Rahul Bahl, Samay Mehta, Henry Seligman, Christopher A. Rajkumar, Suraya Gafore, Sayan Sen, Sukhjinder S. Nijjer, Rasha Al-Lamee, Daniel Chamié, Daniel Bandeira, Xiaowei Zhou, Matthew Shun Shin, Meng-Xing Tang, Ricardo Petraco

**Affiliations:** a Faculty of Medicine, National Heart and Lung Institute, Imperial College London, London, United Kingdom; b Section of Cardiovascular Medicine, Department of Internal Medicine, Yale School of Medicine, New Haven, Connecticut; c Ultrasound Lab for Imaging and Sensing, Department of Bioengineering, Imperial College London, London, United Kingdom

**Keywords:** angina with non-obstructed coronary arteries, coronary flow reserve, coronary microvascular dysfunction, coronary physiology, microvascular angina

## Abstract

**Background:**

Coronary microvascular function assessment with invasive Doppler-derived coronary flow reserve (CFR) has challenges related to Doppler signal quality, limiting clinical adoption. We investigated whether the use of ultrasound microbubble contrast agents can improve coronary flow signals and reduce measurement variability during invasive CFR measurement.

**Methods:**

Participants underwent assessment of coronary flow velocity using a Doppler sensor-tipped coronary wire. Baseline signal quality was determined by expert clinicians, and signals were categorized into good or poor quality. Doppler quality was then assessed at predetermined time periods after administration of SonoVue (Bracco): baseline, first pass, and subsequent discrete 20-second time periods, following this up to 260 seconds after first pass. For each time period, several parameters were assessed: signal-to-noise ratio, Doppler envelope quality (analyzed by a previously validated artificial intelligence–based score), and the coefficient of variation of tracked flow velocity.

**Results:**

Fifteen cases were included. In the group with poor quality baseline Doppler signal (N = 10), signal-to-noise ratio, Doppler envelope quality score, and coefficient of variation significantly improved with microbubble contrast (signal-to-noise ratio: *P* < .05 for all periods after first pass; Doppler envelope quality score: *P* < .05 for all periods; coefficient of variation: *P* < .05 for all periods). In the group with a good quality baseline Doppler signal (N = 5), there were no changes in these parameters. Microbubble administration did not cause significant coronary hyperemia (*P* = .875 when compared to baseline).

**Conclusions:**

Ultrasound microbubble contrast agents can enhance Doppler signal quality during invasive CFR measurements. These agents could be used to improve the reliability of flow measurements in clinical practice and facilitate the adoption of coronary microvascular assessment.

## Introduction

Assessment of coronary microvascular function and coronary flow reserve (CFR) or microvascular resistance with invasive Doppler is a guideline-recommended methodology to investigate symptoms and prognosis in patients with coronary artery disease.^1^, ^2^, ^3^ The main barrier for its wider adoption is the acknowledged technical challenges of invasive Doppler measurements.^4^, ^5^, ^6^, ^7^

We have recently developed an artificial intelligence (AI)-based tool capable of quantifying Doppler signal quality and improving Doppler envelope digital tracking.^8^ However, the issue remains that in some cases, Doppler signals are simply too faint or noisy to be reliably tracked even with AI-based tools. In research studies, between 13% and 31% of attempted Doppler-derived microvascular assessments are not possible.^9^, ^10^, ^11^ Challenges occur for a range of reasons, including a suboptimal or unstable wire sensor location, difficulty manipulating the Doppler sensor, and turbulent coronary blood flow, for example, due to atherosclerotic plaque or vessel tortuosity. Enhancing the raw Doppler signal density could facilitate its digitization and derivation of CFR or resistance, potentially increasing the reliability of coronary microvascular assessment in clinical practice.

Microbubble-based contrast agents are clinically approved for use to enhance noninvasive ultrasound Doppler signal intensity in peripheral, cerebral, and extracranial arteries as well as liver and breast lesions.^12^ They are also recommended in multiple international echocardiography guidelines for improving the delineation of the endocardial border, enabling more accurate measurement of ventricular function and thrombus identification.^13^, ^14^, ^15^, ^16^ Despite their established role in noninvasive ultrasound-based modalities, the effects of microbubble contrast on invasive Doppler signals have never been formally tested. In this study, we investigate the effects of a microbubble ultrasound contrast agent on invasive coronary flow Doppler signal quality.

## Materials and methods

### Study design and protocol

Participants were recruited from 1 of 3 ongoing trials involving the use of Doppler-derived coronary flow velocity for microvascular function assessment as part of the research protocol. All participants gave written informed consent. Ethical approval was obtained from the local research ethics committee (reference numbers: 18/LO/1998, 20/LO/1166, 19/LO/1194).

Ten participants with poor quality Doppler signals and 5 with good quality Doppler signals, as judged by the operator, were included. Poor quality Doppler signals were considered to be those where the Doppler envelope was faint or noisy, leading to poor tracking of the flow velocity, whereas good quality Doppler signals were considered to be those with a dense Doppler envelope easily tracked by the console. Participants were selected and allocated to these groups prior to observing the effect of microbubble ultrasound contrast. All appropriate attempts were made to achieve an optimal Doppler signal through repositioning of the sensor wire. The coronary vessel interrogated was determined by the respective trial protocol. Participants with anatomy unsuitable for physiological assessment were excluded. Microvascular assessments were made in unobstructed coronary arteries or after PCI in cases where PCI was performed. Unobstructed arteries were defined according to angiographic assessment and physiological interrogation (fractional flow reserve >0.8), where any angiographically moderate or severe stenosis was present. All participants provided written informed consent and were included in the analysis.

### Procedures

Doppler recordings were obtained using a 0.014-inch intravascular steerable Doppler sensor-tipped guide wire (Combowire XT, Philips) delivered to a coronary artery via a 6F guide catheter, after administration of full anticoagulation and intracoronary nitrates. The guide wire was placed in 1 of the 3 major epicardial coronaries. The ComboWire XT tip was gently manipulated, avoiding bends or knuckles to obtain a stable and optimal Doppler signal. The SonoVue contrast was prepared as per the manufacturer’s guidelines by adding 5 mL of normal saline to the vial and thoroughly shaking the preparation immediately before administration. One milliliter of the reconstituted contrast was delivered intravenously, followed by 10 mL of normal saline.

SonoVue contrast consists of inert gas (sulfur hexafluoride) microbubbles averaging 2.5 μm in diameter, encapsulated within a phospholipid shell that provides stability to the microbubbles, preventing them from coalescing or dissolving in the bloodstream. The microbubbles are highly echogenic, which enhances Doppler and ultrasound signals. They persist in the bloodstream for several minutes, allowing for real-time assessment of invasive Doppler signals.

### Analysis

#### Signal quality and time period categorization

**Figure 1:**
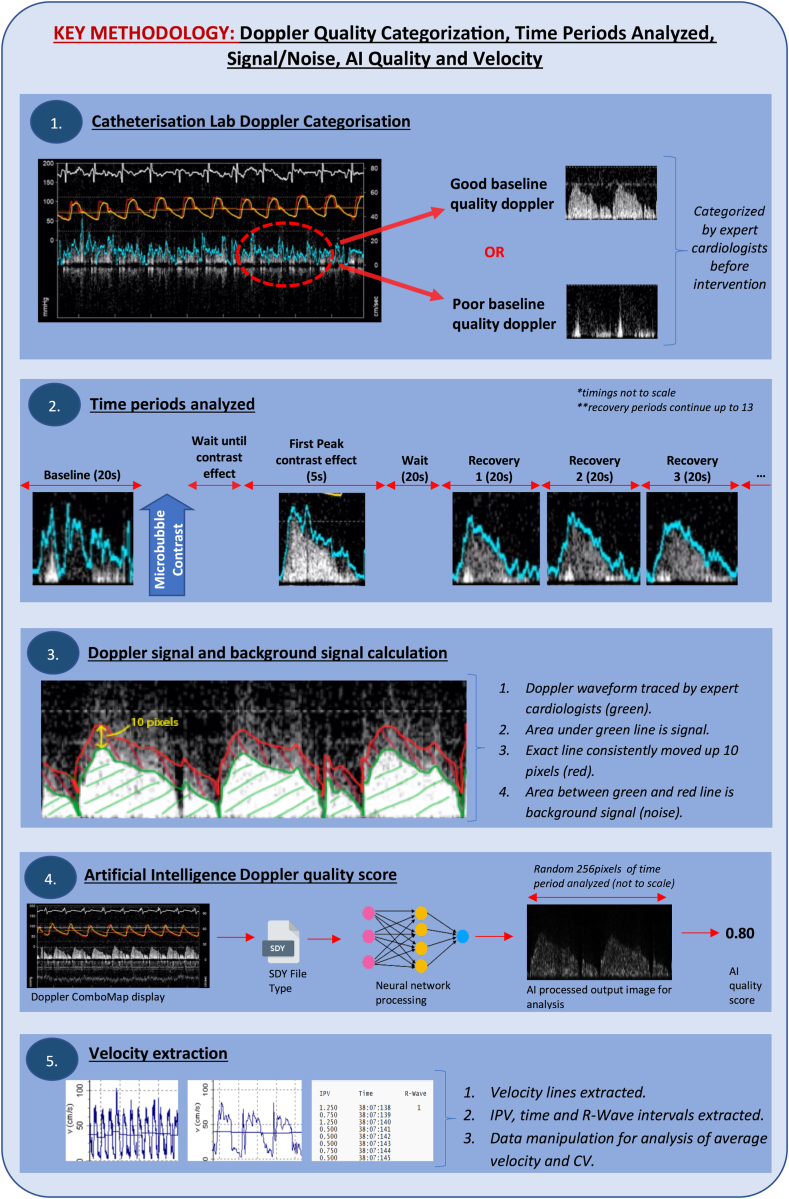
**Summary of the study methodology**. AI, artificial intelligence; CV, coefficient of variation; IPV, instantaneous peak velocity.

The Doppler signal quality was assessed by 2 cardiologists experienced in the measurement of coronary flow velocity and categorized into “good” or “poor” signal quality (Figure 1, panel 1). Effects were assessed separately in each group.

Data were analyzed for up to 14 different time periods for each participant. These included 20 seconds before contrast administration (labeled “Before Injection”), a 5-second interval when the contrast had its first and maximum effects (labeled “First Pass”), and then after a delay of 20 seconds, up to 12 consecutive 20-second periods (Figure 1, panel 2).

#### Signal-to-noise ratio

For each time period, an expert cardiologist manually traced the Doppler envelope for the first 10 consecutive beats. Custom software was used to duplicate and move this line upward by 10 pixels. The average grayscale color (ranging from 255 for pure white and 0 for pure black) was determined for the area under the manually traced line (signal) and the area between the 2 lines (noise). The ratio between the 2 was calculated as the signal-to-noise ratio (Figure 1, panel 3).

#### Doppler envelope quality score

Doppler envelope images for each time period were analyzed and scored by a previously validated neural network^8^ (Figure 1, panel 4).

#### Measured flow velocity and variation in measured velocity

The individual numerical average peak velocity values were extracted for each time period using Study Manager (Volcano Corp). Average velocity and coefficient of variation were then calculated using custom software in MATLAB (MathWorks Inc) (Figure 1, panel 5). The coefficient of variation, defined as the ratio of the standard deviation to the mean, was used to quantify the extent of variability within the data. The coefficient of variation was not calculated for the “First Pass” time period, as this is a shorter duration, which precluded direct comparison with the other periods. Mean flow velocity was reported only for those with good signal quality before injection because this was the only group where the flow velocity could be reliably measured at this time point.

#### Statistical analysis

Continuous variables are reported as median ± interquartile range. Categorical variables are reported as numbers and proportions. For each variable, a paired Wilcoxon signed-rank test was used to compare the preinjection value with each subsequent time period, up to 140 seconds after “First Pass.” The Holm-Bonferroni method was used to adjust for multiple comparisons. Beyond 140 seconds, statistical significance was not assessed due to insufficient data.

An additional analysis was performed to evaluate Doppler quality before contrast as a continuous variable. A generalized least squares linear model was fitted, predicting the Doppler envelope quality score according to time from contrast administration and the Doppler envelope quality score before contrast.

A 2-tailed *P* value <.05 was considered statistically significant. All statistical analyses were performed using R version 4.2.3 (R Foundation for Statistical Computing).

## Results

**Figure 2:**
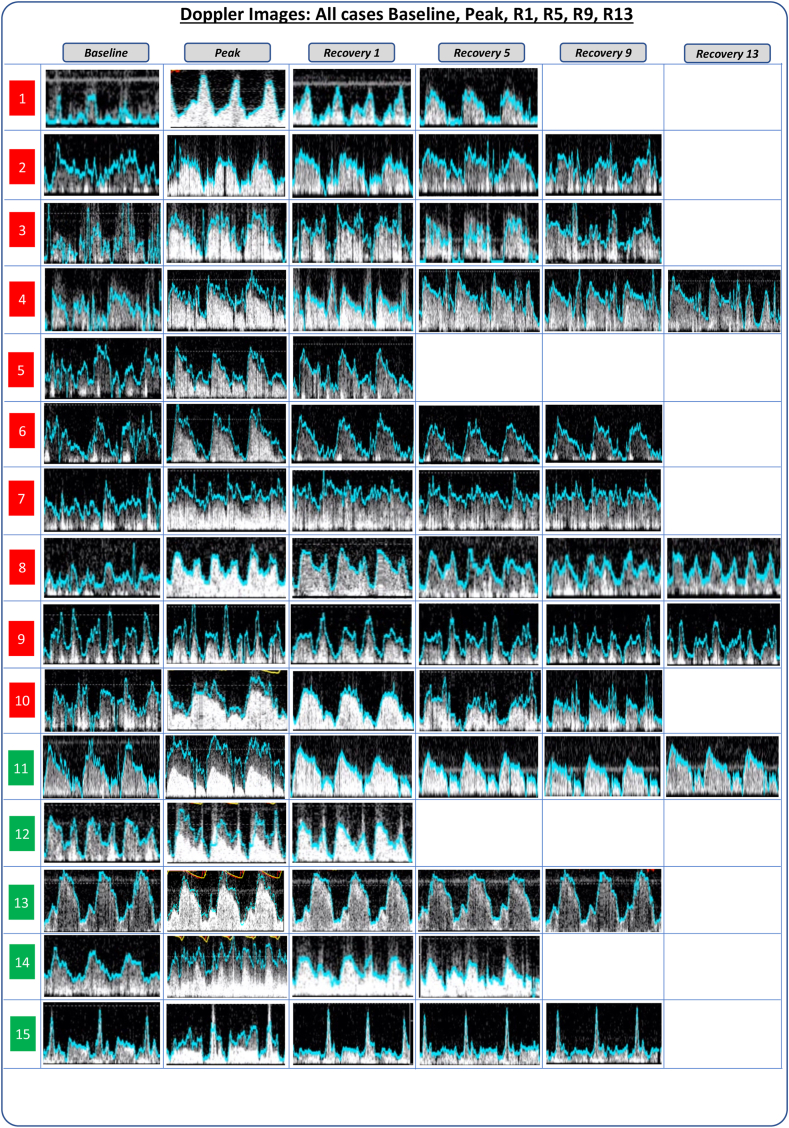
**Doppler images of all included vessels demonstrating the effect of microbubble ultrasound contrast.** The first 10 rows (red numbers) show those judged to have poor quality signals, whereas the next 5 rows (green numbers) show those judged to have good quality signals. After the administration of microbubble contrast, one can appreciate an improvement in the quality of the Doppler signal, with a marked improvement in signal density. In addition, particularly in the group with poor quality signals, there is a marked reduction in the variability of the tracked velocities (light blue line).

Demographic data of patients are summarized in Table 1. Doppler and flow velocity tracking of all 15 cases included (10 with poor baseline Doppler quality and 5 with good baseline Doppler quality) are shown in Figure 2. One case from the good quality group had hyperemia induced during the measurement period and was therefore excluded from the analysis of tracked velocity and velocity variability.

**Table 1:** Baseline characteristics.

Characteristic	Baseline Doppler signal quality	
	Good (n = 5)	Poor (n = 10)
Age, y	55 (46-59)	63 (55-68)
Sex		
Male	4 (80%)	6 (60%)
Female	1 (20%)	4 (40%)
Body mass index, kg/m^2^	24.1 (19.7-26.8)	25.5 (22.7-30.3)
Smoking status		
Nonsmoker	2 (40%)	6 (60%)
Ex-smoker	1 (20%)	3 (30%)
Smoker	2 (40%)	1 (10%)
Hypertension	2 (40%)	4 (40%)
Diabetes	2 (40%)	4 (40%)
Hyperlipidemia	1 (20%)	6 (60%)
eGFR, mL/min/1.73 m^2^	90 (29-90)	86 (75-90)
Previous PCI	1 (20%)	0 (0%)
Previous MI	2 (40%)	0 (0%)
Left ventricular ejection fraction, %	52 (28-60)	58 (55-60)
Indication		
Angina	0 (0%)	4 (40%)
Anomalous coronary artery	3 (60%)	4 (40%)
Heart failure	2 (40%)	2 (20%)
Procedure type		
Diagnostic	4 (80%)	8 (80%)
PCI	1 (20%)	2 (20%)
Vessel		
Left anterior descending artery	2 (40%)	7 (70%)
Right coronary artery	3 (60%)	3 (30%)
Coronary artery disease		
Multivessel	1 (20%)	2 (20%)
Single vessel	0 (0%)	1 (10%)
Unobstructed	4 (80%)	7 (70%)

Values are median (IQR) or n (%). eGFR, estimated glomerular filtration rate; MI, myocardial infarction; PCI, percutaneous coronary intervention.

### Effect of contrast agent on signal-to-noise ratio of Doppler envelopes

**Figure 3:**
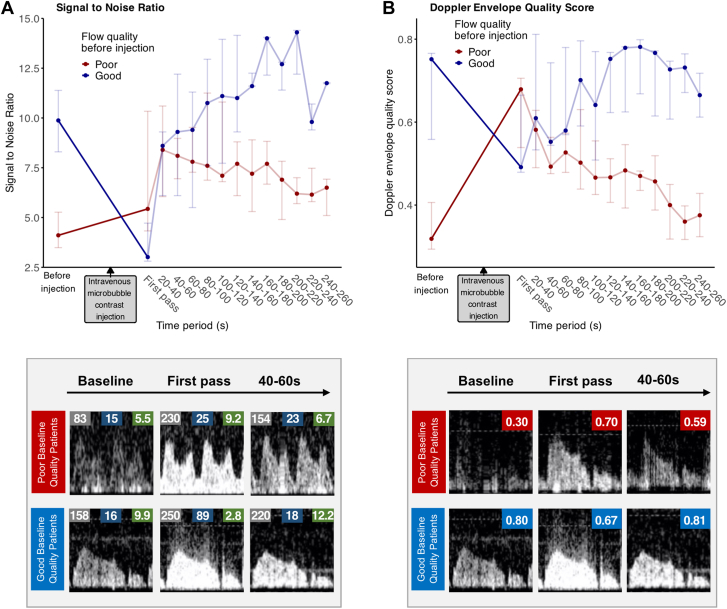
**Effect of microbubble contrast on Doppler signal quality.** Signal-to-noise ratio (**A**) and Doppler envelope quality score (**B**). Points and error bars represent the median and interquartile range, respectively. Representative images are displayed in the lower figures. For signal-to-noise ratio (**A**, lower), the gray number is signal, the blue number is noise, and the green number is signal-to-noise ratio. For Doppler envelope quality score (**B**, lower), the number is the Doppler envelope quality score.

Changes in the Doppler signal-to-noise ratio are presented in Figure 3A and Supplemental Table S1. In the poor quality Doppler signal group, the signal-to-noise ratio increased significantly at 20 to 40 seconds after first pass and then remained significantly greater than preinjection until 140 seconds, (before injection: 4.11 ± 1.78; first pass: 5.43 ± 6.01, *P* = .0131; 20-40 seconds: 8.4 ± 4.55, *P* = .049; 40-60 seconds: 8.1 ± 2.02, *P* = .041; 60-80 seconds: 7.8 ± 2.25, *P* = .049; 80-100 seconds: 7.6 ± 4.38, *P* = .049; 100-120 seconds: 7.1 ± 4, *P* = .047; 120-140 seconds: 7.7 ± 2.7, *P* = .049).

In the good quality Doppler signal group, there was no significant difference in signal-to-noise ratio when compared to baseline (before injection, 9.88 ± 3.08; first pass, 3.01 ± 1.91, *P* = .438; 20-40 seconds: 8.6 ± 3.2, *P* > .99; 40-60 seconds: 9.3 ± 6.1, *P* > .99; 60-80 seconds: 9.4 ± 5.8, *P* > .99; 80-100 seconds: 10.75 ± 5.35, *P* > .99; 100-120 seconds: 11.1 ± 6.23, *P* > .99; 120-140 seconds: 11 ± 4.85, *P* > .99).

**Supplementary Figure S1:**
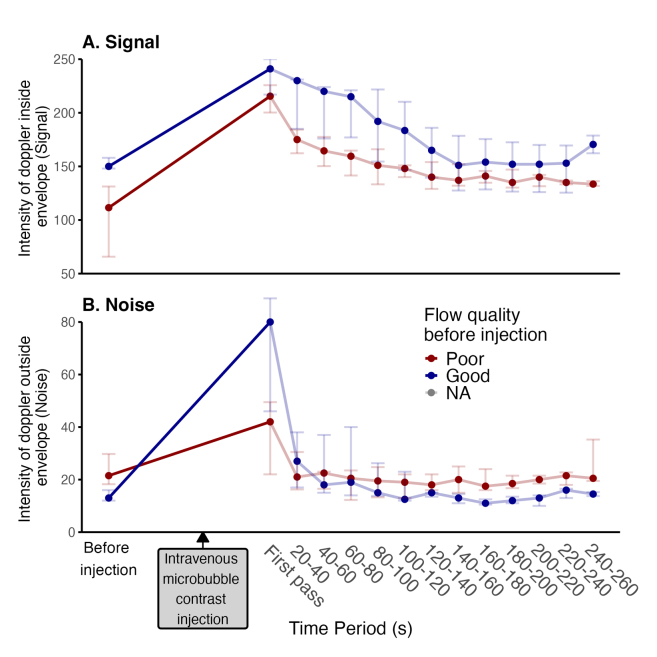


Supplemental Figure S1 displays the signal and noise parameters separately. In both groups, the Doppler envelope signal increased with contrast administration. There was a numerically greater increase in noise outside the Doppler envelope at first pass in the good quality Doppler signal group. Following this, there was no difference in noise in either group when compared to baseline.

### Effect of contrast agent on Doppler envelope quality score

Our previously expert-validated AI-based score was used to quantify the changes in Doppler signal quality caused by microbubble contrast. Results are displayed in Figure 3B and Supplemental Table S2. In the good quality Doppler signal group, there were no significant differences compared to preinjection values at any subsequent postcontrast time period (before injection: 0.75 ± 0.21; first pass: 0.49 ± 0.19, *P* = .438; 20-40 seconds: 0.61 ± 0.28, *P* > .99; 40-60 seconds: 0.55 ± 0.2, *P* > .99; 60-80 seconds: 0.58 ± 0.21, *P* > .99; 80-100 seconds: 0.7 ± 0.21, *P* > .99; 100-120 seconds: 0.64 ± 0.26, *P* > .99; 120-140 seconds: 0.75 ± 0.15, *P* > .99). In the poor quality Doppler signal group, there was a statistically significant improvement in the Doppler envelope quality score at all subsequent time periods until 140 seconds (before injection: 0.32 ± 0.11; first pass: 0.68 ± 0.17, *P* = .014; 20-40 seconds: 0.58 ± 0.14, *P* = .014; 40-60 seconds: 0.49 ± 0.09, *P* = .014; 60-80 seconds: 0.53 ± 0.09, *P* = .014; 80-100 seconds: 0.5 ± 0.19, *P* = .014; 100-120 seconds: 0.47 ± 0.13, *P* = .014; 120-140 seconds: 0.47 ± 0.11, *P* = .014).

There was an inverse relationship between the percentage change in Doppler envelope quality and the baseline quality. The greatest improvements were seen in traces with the poorest baseline quality, whereas traces with the highest quality showed no improvement or slight deterioration (Supplemental Figure S2).

### Effect of contrast agent on variability of velocity tracking

**Figure 4:**
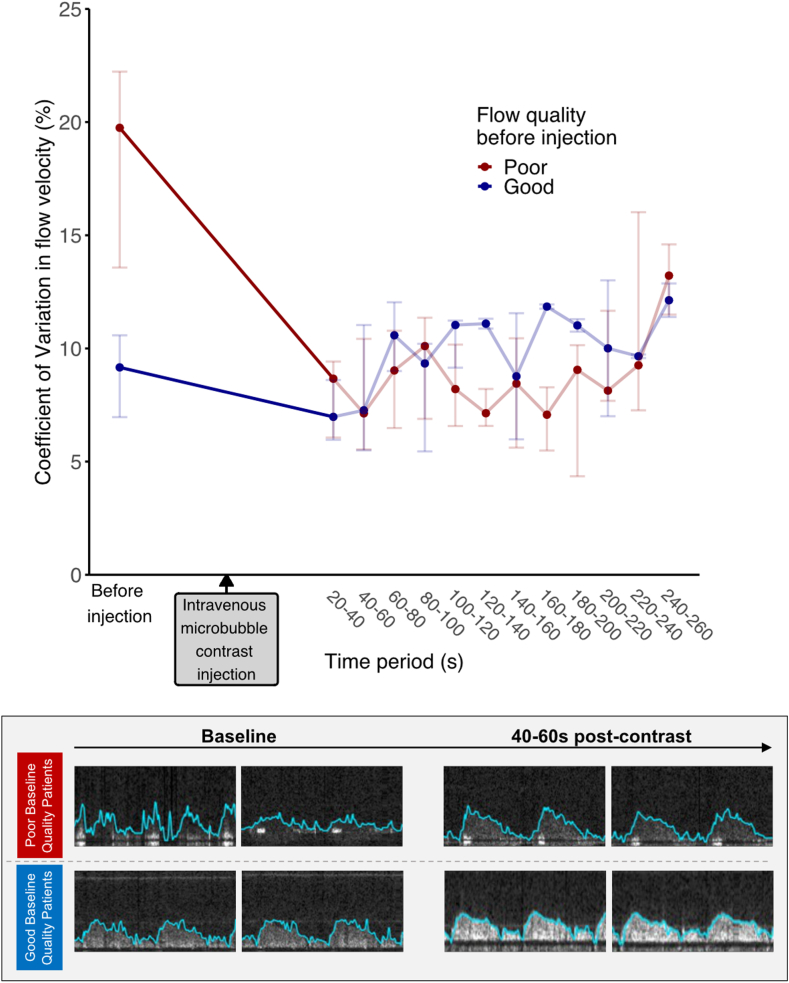
**Coefficient of variation of flow velocity.** Points and error bars represent the median and interquartile range, respectively. Representative images are displayed in the lower figure.

The coefficient of variation of the tracked flow velocity is presented in Figure 4 and Supplemental Table S3. In the good quality Doppler signal group, there was no significant difference when compared to preinjection in any subsequent time periods until 140 seconds (before injection: 9.16 ± 3.62; 20-40 seconds: 6.98 ± 2.64, *P* > .99; 40-60 seconds: 7.27 ± 5.54, *P* = 1.0; 60-80 seconds: 10.58 ± 3.04, *P* > .99; 80-100 seconds: 9.34 ± 4.76, *P* > .99; 100-120 seconds: 11.04 ± 2.08, *P* > .99; 120-140 seconds: 11.09 ± 0.43, *P* > .99).

In the poor quality Doppler signal group, there was a statistically significant reduction in coefficient of variation with contrast injection in all subsequent time periods until 140 seconds (before injection: 19.75 ± 8.66; 20-40 seconds: 8.66 ± 3.36, *P* = .039; 40-60 seconds: 7.13 ± 4.89, *P* = .023; 60-80 seconds: 9.03 ± 4.3, *P* = .039; 80-100 seconds: 10.1 ± 4.47, *P* = .039; 100-120 seconds: 8.2 ± 3.6, *P* = .039; 120-140 seconds: 7.14 ± 1.64, *P* = .039).

### Effects of contrast injection on flow velocity values

**Figure 5:**
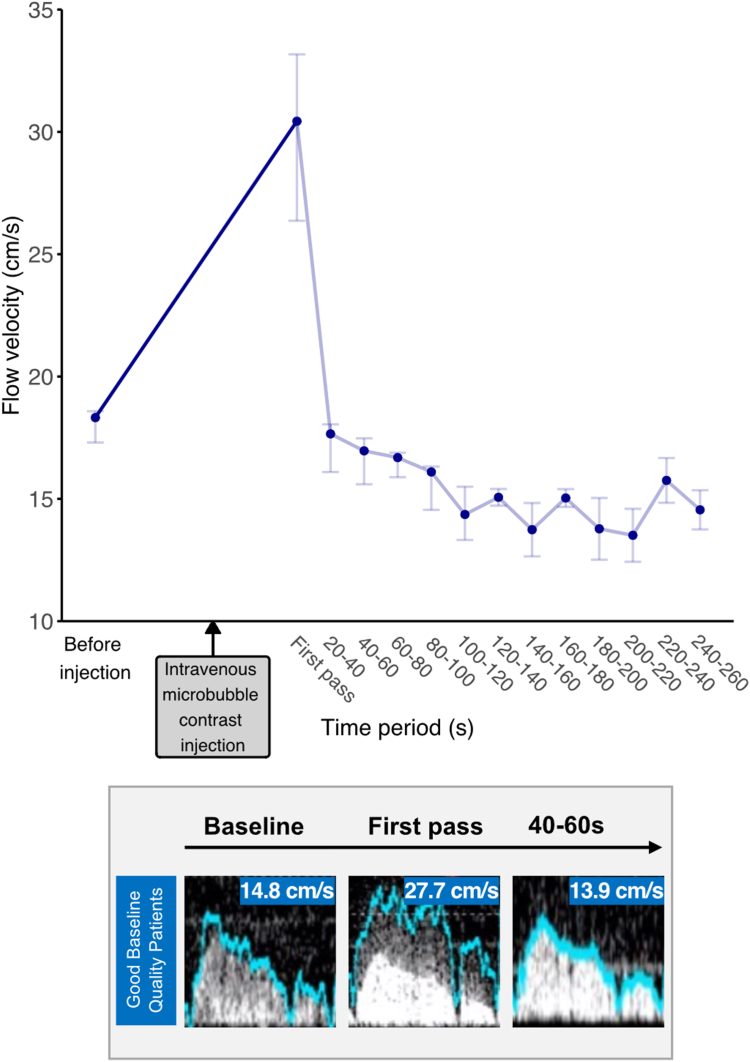
**Flow velocity.** Points and error bars represent the median and interquartile range, respectively. Representative images are displayed in the lower figure.

In those cases where Doppler signal quality was already acceptable at baseline, changes in flow velocity due to contrast injection are presented in Figure 5 and Supplemental Table S4. There was a numerical, but not statistically significant, increase in flow velocity at first pass. There was no difference in flow velocity for all subsequent time periods up to 140 seconds (before injection: 18.32 ± 1.27 cm/s; first pass: 30.43 ± 6.8 cm/s, *P* = .875; 20-40 seconds: 17.66 ± 1.95 cm/s, *P* = .875; 40-60 seconds: 16.96 ± 1.88 cm/s, *P* = .875; 60-80 seconds: 16.69 ± 1 cm/s, *P* = .875; 80-100 seconds: 16.1 ± 1.77 cm/s, *P* = .875; 100-120 seconds: 14.36 ± 2.17 cm/s, *P* = .875; 120-140 seconds: 15.06 ± 0.68 cm/s, *P* = .875).

When examining the Doppler images (Figure 2), it becomes notable that the increase in velocity at first pass is caused by overtracking of the initial noise caused by microbubble contrast bursting.

## Discussion

This study found that the use of the microbubble ultrasound contrast agent significantly increases the quality of invasive coronary Doppler flow signals. Improvements were observed in Doppler signal density, signal-to-noise profile, and quality score. As a result of denser and higher contrast Doppler signals, microbubble contrast administration led to reduced variability and increased precision of flow velocity software tracking (Central Illustration). Notably, these benefits were limited to cases with poor quality Doppler signals, with the greatest benefits observed in the lowest quantiles of signal quality. Conversely, the use of the microbubble contrast agent had negligible or even detrimental effects when the Doppler signal quality was already high.

### Microbubble contrast and Doppler signal intensity

Microbubble contrast increases the signal intensity of ultrasound imaging because the microbubble structure resonates in response to the acoustic waves, making them thousands of times more reflective than normal body tissues.^17^ These agents are widely used to perform contrast-enhanced echocardiography and have also been investigated in several vascular diagnostic techniques, including peripheral, cerebral, and extracranial arteries as well as liver and breast lesion assessment.^13^, ^14^, ^15^, ^16^ Although the use of microbubble contrast-enhanced imaging to assess myocardial perfusion is an emerging field in echocardiography,^18^^,^^19^ very few studies have investigated how these agents could be used to improve the quality of measuring intravascular blood flow velocity using spectral Doppler.

Previous animal studies have demonstrated that microbubble contrast enhances arterial spectral Doppler signal intensity.^20^^,^^21^ Further work has investigated their use in transcranial Doppler imaging in humans,^22^ and 3 studies demonstrated improvement in signal intensity and diagnostic yield using microbubble contrast.^23^, ^24^, ^25^ In one large study, the noninvasive measurement of CFR was facilitated using microbubble contrast during stress echocardiography.^26^ Measurements were made 10 seconds after peak signal intensity after contrast was administered, implying that the effects of contrast were similar on noninvasively obtained coronary Doppler signals as they were in our study. A further study demonstrates a similar approach with transesophageal echocardiography.^27^

Our findings demonstrate that the administration of intravenous microbubble agents increases coronary Doppler signal density, as evidenced visually (Figure 2) and formally with signal-processing metrics (Figure 3, Supplemental Figure S1). Invasive vascular Doppler studies are unique because measurements are made with no interfering tissues between the emitting probe (intracoronary Combowire) and the reflective microbubbles circulating in the bloodstream. This creates an ideal scenario for signal optimization (Doppler intensity) without the generation of tissue noise.

### Increased precision of coronary flow velocity tracking

Much of the measurement variability observed with Doppler-based modalities, including echocardiography,^28^, ^29^, ^30^ peripheral vascular ultrasound,^31^ and invasive coronary physiological assessment,^32^^,^^33^ arises from the difficulty of tracking the flow velocity envelope and transforming its raw analog signal into digital, quantifiable indices.

When operators encounter poor signal quality during microvascular assessment, there are a limited number of options available to them. Manipulation of the sensor wire to try and achieve a position axial to the direction of blood flow is the mainstay technique, but is not always successful. In addition, we recently developed an AI method capable of quantifying coronary Doppler quality and tracking its velocity better than console software.^8^ Despite such improvements, it remains technically challenging (and sometimes impossible) to track coronary Doppler envelopes when the signal is too faint or when background noise is high.

In this study, we found that administration of intravenous microbubble contrast significantly improved the precision of coronary Doppler flow tracking. This can be appreciated both via formal analysis of flow velocity measurement variability (coefficient of variation, Figures 3 and 4, Supplemental Figure S1) and by visual inspection of Doppler envelope tracking (blue lines on Figure 2). In some cases, coronary Doppler envelopes were virtually absent at baseline before contrast injection, with measurements only becoming possible after contrast-induced signal augmentation (cases 1, 3, and 8, Figure 2).

We found that contrast agent administration does not cause significant hyperemia. A transient increase in flow velocity was detected during the first pass of contrast, due to microbubbles bursting when coming into contact with ultrasound waves.^34^, ^35^, ^36^, ^37^ After the first pass, there was no meaningful increase in flow velocity (Figure 5).

### Clinical implications for coronary microvascular assessment

Our findings could have immediate practical implications for clinical practice. International guidelines recommend coronary microvascular assessment in patients with angina and unobstructed coronary arteries,^38^^,^^39^ and recent evidence demonstrates that those with a reduced CFR have an almost 4-fold increase in the risk of all-cause mortality.^2^ However, in practice, adoption of CFR measurement using Doppler remains low, and in part, this is caused by the widely acknowledged measurement challenges. For instance, in research studies, it was not possible to reliably measure CFR in between 13% and 31% of attempted cases.^9^, ^10^, ^11^

**Figure 6:**
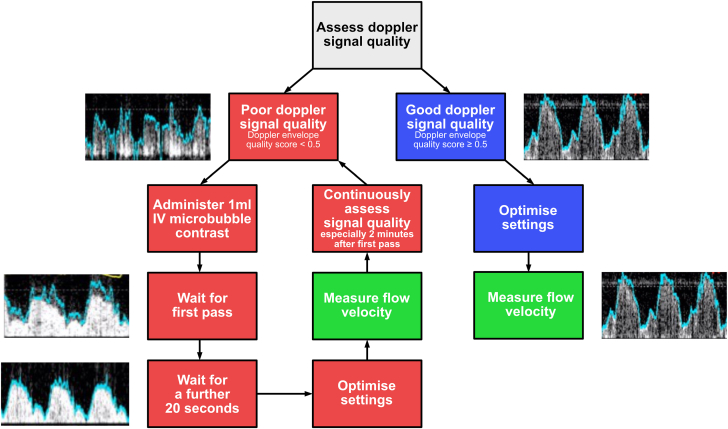
Suggested algorithm for the use of microbubble contrast.

We propose that microbubble contrast agents could be immediately adopted into clinical practice in cases where the coronary Doppler signal is poor or noisy, and we suggest a practical flowchart for their use (Figure 6). Firstly, we recommend only using ultrasound microbubble contrast when the Doppler envelope quality is poor. Determining when the quality is poor with currently available technology requires expert judgment. However, the neural network–derived Doppler envelope quality score would be able to provide an objective assessment if incorporated into console software in the future.^8^ Secondly, the optimal time to conduct measurements of flow velocity when using contrast is between 20 seconds and 140 seconds after the first pass (which is visible a few seconds after its intravenous administration). After this period, if the Doppler envelope quality is no longer acceptable, the process can be repeated until all measurements are complete.

**Central Illustration:**
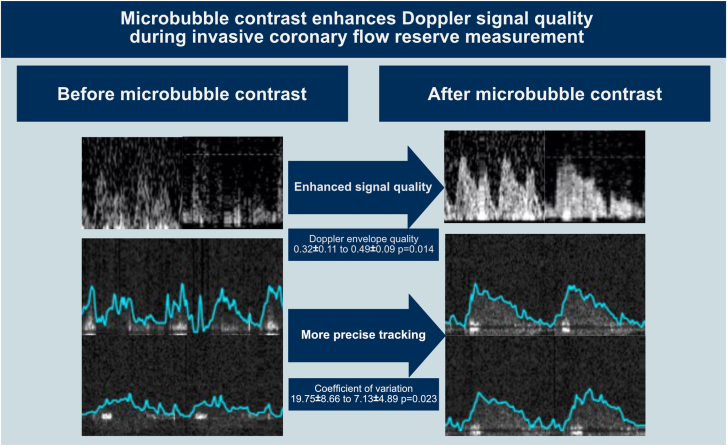
Microbubble contrast improves Doppler signals for coronary microvascular assessment. When the Doppler signal quality is poor, the administration of intravenous microbubble contrast improves the quality of the Doppler signal, and the console is able to track the flow velocity with greater accuracy. No significant improvement is seen when the Doppler quality is already good.

### Limitations

This study has limitations that are important to acknowledge. First, it has a small sample size and, therefore, limited power to detect the effects of contrast in patient subgroups. Second, the assessment of baseline Doppler quality was based on expert judgment rather than an objective measure, and we utilized dichotomous categories. For this reason, we included an additional analysis using Doppler signal quality as a continuous variable. Lastly, only one type of ultrasound microbubble contrast was used, and other formulations may have varying effects on signal quality. Furthermore, in the future, the AI-based Doppler envelope quality score could provide a more objective measure of signal quality before administering microbubble contrast, although this quantification is not available in clinical consoles.

It is difficult to know the true quality of Doppler signals used in previous research studies and in clinical practice for several reasons. The images of the relevant Doppler signals used in calculations are not published in research papers, meaning the data quality cannot be judged in retrospect. In addition, an objective measure quantifying the quality of intracoronary Doppler signals has only recently been developed and hence was not available when previous large data sets were published. Finally, in clinical practice, it is not known how often intracoronary Doppler measurements are attempted but abandoned due to poor signal quality.

Before using microbubble contrast agents, operators must consider their negative effects. Although they are generally safe to administer, there is an approximately 1 in 10000 rate of anaphylactoid reactions, and use of these agents is contraindicated in patients with known allergy or right-to-left shunts.^40^

At the time of this writing, the sensor-tipped guide wire used to gather data for this study (Combowire XT, Philips) is no longer commercially available. We understand that the manufacturer plans to market a new Doppler sensor-tipped guide wire soon. Although it would be reasonable to expect microbubble contrast to have similar effects when using the new guide wire, further work will be needed to demonstrate that our findings still apply to the next generation of Doppler sensor-tipped guide wires.

## Conclusion

Ultrasound microbubble contrast can be used effectively during invasive coronary Doppler flow measurements to improve the Doppler envelope signal and augment the precision of flow velocity tracking. We propose the use of intravenous microbubble contrast during invasive Doppler coronary microvascular assessment when measurements are technically challenging and the Doppler signal is poor or noisy.

## Figures and Tables

**Supplementary Figure S2:**