# Supplementary material for: Microbubble Contrast Agent Use During Invasive Coronary Microvascular Assessment
Source: J Soc Cardiovasc Angiogr Interv. 2025 Jul 31;4(10Part A):103857. doi: 10.1016/j.jscai.2025.103857 (PMC12629735; doi:10.1016/j.jscai.2025.103857)
Supplement: Supplementary Tables S1–S4 [file mmc1.docx]

Tables

Supplementary Table S1: Signal, Noise and Signal to Noise Ratio

Supplementary Table S2: Doppler Envelope Quality Score

Supplementary Table S3: Coefficient of Variation of Flow Velocity

Supplementary Table S4: Flow velocity

### Supplementary Table S1: Signal, Noise and Signal to Noise Ratio

|  | | | | | **Flow quality before injection** | | | | | | |  | | | | | |
| --- | --- | --- | --- | --- | --- | --- | --- | --- | --- | --- | --- | --- | --- | --- | --- | --- | --- |
|  | | **Good** | | | | |  | | | **Poor** | | | |  | | | |
| **Period (s)** | **Signal**  **(average greyscale colour)** | | **Noise**  **(average greyscale colour)** | **Signal to noise ratio** | | **n** | | **p value (SNR)** | **Signal**  **(average greyscale colour)** | | **Noise**  **(average greyscale colour)** | | **Signal to noise ratio** | | **n** | **p value (SNR)** |  |
| Before injection | 150±10 | | 13±10 | 9.88±3.08 | | 5 | |  | 111.5±65.5 | | 21.5±65.5 | | 4.11±1.78 | | 10 |  |  |
| First pass | 241±33 | | 80±33 | 3.01±1.91 | | 5 | | 0.438 | 215.5±25.5 | | 42±25.5 | | 5.43±6.01 | | 10 | 0.131 |  |
| 20-40 | 230±46 | | 27±46 | 8.6±3.2 | | 5 | | 1.000 | 175±22 | | 21±22 | | 8.4±4.55 | | 10 | 0.049 |  |
| 40-60 | 220±48 | | 18±48 | 9.3±6.1 | | 5 | | 1.000 | 164.5±27.25 | | 22.5±27.25 | | 8.1±2.02 | | 10 | 0.041 |  |
| 60-80 | 215±44 | | 19±44 | 9.4±5.8 | | 5 | | 1.000 | 159.5±23.25 | | 20.5±23.25 | | 7.8±2.25 | | 10 | 0.049 |  |
| 80-100 | 192±67.25 | | 15±67.25 | 10.75±5.35 | | 4 | | 1.000 | 151±32.75 | | 19.5±32.75 | | 7.6±4.38 | | 10 | 0.049 |  |
| 100-120 | 183.5±63.5 | | 12.5±63.5 | 11.1±6.23 | | 4 | | 1.000 | 148±11 | | 19±11 | | 7.1±4 | | 9 | 0.047 |  |
| 120-140 | 165±46.5 | | 15±46.5 | 11±4.85 | | 3 | | 1.000 | 140±25 | | 18±25 | | 7.7±2.7 | | 9 | 0.049 |  |
| 140-160 | 151±51 | | 13±51 | 11.6±0.65 | | 3 | |  | 137±20 | | 20±20 | | 7.2±3.6 | | 9 |  |  |
| 160-180 | 154±47 | | 11±47 | 14±1.9 | | 3 | |  | 141±11 | | 17.5±11 | | 7.7±2.15 | | 8 |  |  |
| 180-200 | 152±46 | | 12±46 | 12.7±1.4 | | 3 | |  | 135±16.75 | | 18.5±16.75 | | 6.9±2.92 | | 8 |  |  |
| 200-220 | 152±44 | | 13±44 | 14.3±2.2 | | 3 | |  | 140±10.5 | | 20±10.5 | | 6.2±1.3 | | 7 |  |  |
| 220-240 | 153±44 | | 16±44 | 9.8±1.3 | | 3 | |  | 135±3.5 | | 21.5±3.5 | | 6.15±1.67 | | 6 |  |  |
| 240-260 | 170.5±16.5 | | 14.5±16.5 | 11.75±0.05 | | 2 | |  | 133.5±4.5 | | 20.5±4.5 | | 6.5±1.82 | | 4 |  |  |
| Values are median±interquartile range | | | | | | | | | | | | | | | | |  |

### Supplementary Table S2: Doppler Envelope Quality Score

|  | **Flow quality before injection** | | | | |  |
| --- | --- | --- | --- | --- | --- | --- |
|  | **Good** | |  | **Poor** | |  |
| **Period (s)** | **Doppler envelope quality score** | **n** | **p value** | **Doppler envelope quality score** | **n** | **p value** |
| Before injection | 0.75±0.21 | 5 |  | 0.32±0.11 | 10 |  |
| First pass | 0.49±0.19 | 5 | 0.438 | 0.68±0.17 | 10 | 0.014 |
| 20-40 | 0.61±0.28 | 5 | 1.000 | 0.58±0.14 | 10 | 0.014 |
| 40-60 | 0.55±0.2 | 5 | 1.000 | 0.49±0.09 | 10 | 0.014 |
| 60-80 | 0.58±0.21 | 5 | 1.000 | 0.53±0.09 | 10 | 0.014 |
| 80-100 | 0.7±0.21 | 4 | 1.000 | 0.5±0.19 | 10 | 0.014 |
| 100-120 | 0.64±0.26 | 4 | 1.000 | 0.47±0.13 | 9 | 0.014 |
| 120-140 | 0.75±0.15 | 3 | 1.000 | 0.47±0.11 | 9 | 0.014 |
| 140-160 | 0.78±0.16 | 3 |  | 0.48±0.15 | 9 |  |
| 160-180 | 0.78±0.13 | 3 |  | 0.47±0.05 | 8 |  |
| 180-200 | 0.77±0.16 | 3 |  | 0.46±0.13 | 8 |  |
| 200-220 | 0.73±0.14 | 3 |  | 0.4±0.13 | 7 |  |
| 220-240 | 0.73±0.09 | 3 |  | 0.36±0.08 | 6 |  |
| 240-260 | 0.66±0.11 | 2 |  | 0.38±0.1 | 4 |  |
| Values are median±interquartile range | | | | | | |

### Supplementary Table S3: Coefficient of Variation of Flow Velocity

|  | | **Flow quality before injection** | | | | | | | |  |  |
| --- | --- | --- | --- | --- | --- | --- | --- | --- | --- | --- | --- |
|  | | **Good** | |  | | | **Poor** | | |  |  |
| **Period (s)** | **Coefficient of Variation (%)** | | **n** | | **p value** | **Coefficient of Variation (%)** | | **n** | **p value** | | |
| Before injection | 9.16±3.62 | | 4 | |  | 19.75±8.66 | | 9 |  | | |
| 20-40 | 6.98±2.64 | | 4 | | 1.000 | 8.66±3.36 | | 9 | 0.039 | | |
| 40-60 | 7.27±5.54 | | 4 | | 1.000 | 7.13±4.89 | | 9 | 0.023 | | |
| 60-80 | 10.58±3.04 | | 4 | | 1.000 | 9.03±4.3 | | 9 | 0.039 | | |
| 80-100 | 9.34±4.76 | | 3 | | 1.000 | 10.1±4.47 | | 9 | 0.039 | | |
| 100-120 | 11.04±2.08 | | 3 | | 1.000 | 8.2±3.6 | | 8 | 0.039 | | |
| 120-140 | 11.09±0.43 | | 2 | | 1.000 | 7.14±1.64 | | 8 | 0.039 | | |
| 140-160 | 8.77±5.57 | | 2 | |  | 8.45±4.83 | | 8 |  | | |
| 160-180 | 11.85±0.19 | | 2 | |  | 7.07±2.79 | | 8 |  | | |
| 180-200 | 11.02±0.55 | | 2 | |  | 9.05±5.79 | | 8 |  | | |
| 200-220 | 10.01±6 | | 2 | |  | 8.14±3.98 | | 7 |  | | |
| 220-240 | 9.65±0.16 | | 2 | |  | 9.26±8.76 | | 6 |  | | |
| 240-260 | 12.13±1.48 | | 2 | |  | 13.22±3.1 | | 4 |  | | |
| Values are median±interquartile range | | | | | | | | | | | |

### Supplementary Table S4: Flow velocity

| **Good flow quality before injection** | | | |
| --- | --- | --- | --- |
| **Period (s)** | **Velocity**  **(cm/s)** | **n** | **p** |
| Before injection | 18.32±1.27 | 4 |  |
| First pass | 30.43±6.8 | 4 | 0.875 |
| 20-40 | 17.66±1.95 | 4 | 0.875 |
| 40-60 | 16.96±1.88 | 4 | 0.875 |
| 60-80 | 16.69±1 | 4 | 0.875 |
| 80-100 | 16.1±1.77 | 3 | 0.875 |
| 100-120 | 14.36±2.17 | 3 | 0.875 |
| 120-140 | 15.06±0.68 | 2 | 0.875 |
| 140-160 | 13.74±2.18 | 2 |  |
| 160-180 | 15.03±0.73 | 2 |  |
| 180-200 | 13.78±2.52 | 2 |  |
| 200-220 | 13.51±2.16 | 2 |  |
| 220-240 | 15.75±1.83 | 2 |  |
| 240-260 | 14.55±1.6 | 2 |  |
| Values are median±interquartile range | | |  |
